# Unlocking the potential of microalgae cultivated on wastewater combined with salinity stress to improve biodiesel production

**DOI:** 10.1007/s11356-023-30370-6

**Published:** 2023-10-21

**Authors:** Mohamed E. H. Osman, Atef M. Abo-Shady, Saly F. Gheda, Samy M. Desoki, Mostafa E. Elshobary

**Affiliations:** Botany Department, Faculty of Science, Tanta University, Tanta, 31527 Egypt

**Keywords:** *Oocystis pusilla*, Muncible wastewater, Total dissolved solids, Lipid productivity, Fatty acid profile, Biofuel

## Abstract

Microalgae have the potential as a source of biofuels due to their high biomass productivity and ability to grow in a wide range of conditions, including wastewater. This study investigated cultivating two microalgae species, *Oocystis pusilla* and *Chlorococcus infusionum*, in wastewater for biodiesel production. Compared to Kühl medium, KC medium resulted in a significant fold increase in cellular dry weight production for both *O. pusilla* and *C. infusionum*, with an increase of 1.66 and 1.39, respectively. A concentration of 100% wastewater resulted in the highest growth for *O. pusilla*, with an increase in biomass and lipid content compared to the KC medium. *C. infusionum* could not survive in these conditions. For further increase in biomass and lipid yield of *O. pusilla*, different total dissolved solids (TDS) levels were used. Maximum biomass and lipid productivities were achieved at 3000 ppm TDS, resulting in a 28% increase in biomass (2.50 g/L) and a 158% increase in lipid yield (536.88 mg/g) compared to KC medium. The fatty acid profile of *O. pusilla* cultivated on aerated wastewater at 3000 ppm TDS showed a high proportion of desirable saturated fatty acids (SFA) and monounsaturated fatty acids (MUFA) for biodiesel production. Cultivating microalgae in wastewater for biodiesel production can be cost-effective, especially for microalgae adapted to harsh conditions. It could be concluded that *O. pusilla* is a promising candidate for biodiesel production using wastewater as a growth medium, as it has high biomass productivity and lipid yield, and its fatty acid profile meets the standard values of American and European biodiesel standards. This approach offers a sustainable and environmentally friendly solution for producing biofuels while reducing the environmental impact of wastewater disposal.

**Supplementary Information:**

The online version contains supplementary material available at 10.1007/s11356-023-30370-6.

## Introduction

It is well-known that the utilization of conventional fuels in energy generation has widely acknowledgedly led to a range of environmental issues, including air pollution, the release of greenhouse gases, and the onset of climate change. Fossil fuels are finite resources that are being depleted rapidly, and their extraction and use significantly impact local communities and ecosystems. In addition, the geopolitical implications of fossil fuel dependence have led to conflicts and geopolitical instability (Abdelsalam et al. 2019; Abomohra and Elshobary 2019). Therefore, there is an immediate requirement to shift towards renewable and sustainable energy sources to mitigate these environmental and social issues. Microalgae-based biodiesel production has the potential to be a sustainable and eco-friendly alternative to conventional fuels, as it can be produced using renewable resources such as sunlight and wastewater and has a lower carbon footprint compared to fossil fuels (EL-Seesy et al. 2021; Chandrasekhar et al. 2022). By promoting the development of biomass-based biodiesel production, we can take a step towards reducing our reliance on conventional fuels and addressing the environmental and social problems associated with their use.

Algae have gained significant attention as a potential in everything from food, feed, bio-energy, pharmaceutical, and cosmetics (El‐Khodary et al. 2020; Siddiki et al. 2022; Huo et al. 2022; El-Sapagh et al. 2023). Because of their substantial lipid content, swift growth rate, and ability to flourish in various settings, microalgae have garnered significant attention as a feasible option for biodiesel production (Venkata Subhash et al. 2022; Ali et al. 2023). The high cost of microalgae cultivation and the low biomass productivity are two significant obstacles to commercializing microalgae-based biodiesel production. Cultivating microalgae requires substantial resource allocation, including water, nutrients, and energy, which can incur significant costs.

An encouraging strategy to cut down on the expenses associated with microalgae cultivation involves employing wastewater as a growth medium. Wastewater is a cheap and abundant resource that can provide nutrients and water for microalgae growth. In addition, using wastewater for microalgae cultivation can help to reduce environmental pollution by treating the wastewater and recovering valuable resources (Elshobary et al. 2019; Wang et al. 2023a, b). Numerous studies have consistently demonstrated that the utilization of wastewater yields not only the benefit of wastewater recycling but also presents a significant opportunity for biomass conversion and production. This phenomenon is markedly pronounced in the context of microalgae-based biofuel production and various other applications, all of which hold exceptional promise. Microalgae cultivated in wastewater can amass lipids, varying from minimal levels at 10% of dry weight to more substantial amounts at 25–30% of dry weight, accounting for > 60% of their dry weight (Abou-Shanab et al. 2013). Algae, owing to their lipid accumulation capacity and robust photosynthetic productivity, exhibit a yearly oil yield that surpasses palm oil by a significant margin, ranging from 7 to 31 times higher (Hu et al. 2008; El-Sheekh et al. 2017). Biodiesel derived from microalgae represents a potential sustainable energy source capable of substituting fossil fuels, all while safeguarding the availability of food resources for human consumption (Enamala et al. 2018; Elshobary et al. 2022). While microalgae have demonstrated potential as a feedstock for biodiesel manufacture, the challenges of selecting a suitable microalgal species, enhancing growth rate, and stimulating lipid accumulation still exist. Choosing an appropriate algal strain is paramount for efficient biodiesel production, as different strains vary in their lipid content, growth rate, and tolerance to environmental stressors. Enhancing the growth rate of microalgae can also be challenging, as it requires optimizing various growth factors such as temperature, pH, light intensity, and nutrient availability. Additionally, motivating cellular lipid synthesis and accumulation in algae requires the application of specific stressors or genetic modifications to alter the metabolic pathways of algae (Barakat et al. 2021). Attempts are underway to address these encounters and develop more efficient and sustainable microalgae-based biodiesel production methods. For example, mutation techniques can modify microalgae's metabolic pathways to increase their lipid content and enhance their growth rate (Elshobary et al. 2022). Additionally, using low-cost and sustainable growth media such as wastewater and developing efficient lipid extraction methods can help lower the total cost of biodiesel production from microalgae (Abomohra and Elshobary 2019; Wang et al. 2023b). While the high costs of cultivation and extraction have been a major barrier, recent techno-economic analyses suggest the potential for commercially viable microalgae biodiesel production through optimized processes and scaled-up systems to reduce costs (Xin et al. 2016).

To further enhance the lipid content in microalgae, various stress environments such as nutrient deficit (Pandit et al. 2017) as well as salinity stress (measured by total dissolved solids or TDS) can be applied during cultivation. The application of salinity stress to enhance lipid production in microalgae has been broadly investigated and applied in the production of biodiesel (Paliwal et al. 2017; Abomohra et al. 2020) for freshwater microalgae such as *Chlorella* sp*.* (Yun et al. 2019) or even marine species *Dunaliella* (BenMoussa-Dahmen et al. 2016), *Tetraselmis* sp*.* and *Nannochloropsis* sp*.* (Khatoon et al. 2014). Salinity stress is a common environmental stressor that can be easily controlled during microalgae cultivation. It involves exposing microalgae to high salt concentrations, which can alter their physiology and metabolism, leading to the accumulation of lipids. In this regard, microalgae have evolved distinct mechanisms to adjust to salinity stress. These mechanisms may include the accumulation of osmo-protective solutes, the production of antioxidant enzymes, the regulation of ion exchange processes, and a shift from active cell division to energy storage in the form of lipids (Talebi et al. 2013). The degree of lipid accumulation varies depending on the salinity levels and the microalgae species. Salinity stress could potentially be problematic for certain types of microalgae, given that each strain has a unique ideal salinity level for both growth and lipid accumulation. Thus, the examination of microalgae to identify a species with high lipid content across a broad range of salinity levels is crucial. While some studies have explored microalgae cultivation on wastewater, there is a limited understanding of how different microalgae species respond to 100% wastewater and increasing salinity levels (Elshobary et al. 2019). This study aimed to fill this knowledge gap by evaluating the biomass and lipid productivity potential of two new native strains that are naturally adapted to grow in the wastewater environment available locally, microalgae species, *Oocystis pusilla* and *Chlorococcus infusionum*, grown on 100% wastewater and with varying total dissolved solids (TDS) levels. We demonstrate the feasibility of using wastewater to unlock the potential of *O. pusilla* for biodiesel production through significantly increased biomass and lipid yields. This innovative approach could provide a cost-effective and environmentally sustainable solution for microalgal biofuel production.

## Materials and methods

### Microalgae isolation

Two microalgae species were isolated from the aerated bond muncible wastewater (AWW) treatment plant located in ESH ELMALAHA of Hurghada, Egypt (25°04′10.0″N 34°53′15.2″E). The wastewater samples were transported to the laboratory and cultured into a 10% Kühl medium (Kühl and Lorenzen 1964) at room temperature (25 ± 3 °C) for a week under continuous illumination of cool fluorescent lamps (40 µmol m^−2^s^−1^; Philips Co., Taipei, Taiwan). Following a visual assessment of algal growth, 20 μL of the culture was streaked onto a solid Kühl medium containing 2% agar. To acquire pure cultures, the green algae were isolated and purified through the sub-culturing technique. The microalgae cells were examined microscopically (Olympus BH2, Olympus, Shinjuku-ku, Tokyo, Japan). Algal morphological characters were examined, and images of algal structures were captured, encompassing their form, dimensions, and cellular features such as the flagella, chloroplast, and cell wall. These isolated microalgae were classified by referring to previously documented findings (Smith 1950; Hendy 1964; Prescott 1987). The isolated algae were conserved on Kühl (1.5%) medium agar slants at 4 °C for future work.

### Wastewater characterization

Water samples were collected from the aerated bond wastewater (AWW) treatment plant located in ESH ELMALAHA of Hurghada, Egypt, at geographic coordinates 25°04′10.0″N 34°53′15.2″E. In situ wastewater temperature, electrical conductivity, and pH measurements were monitored using the Hydrolab Model (Multi Set 430i WTW). Secchi-disk was used to determine transparency (diameter 30 cm). Alkalinity, chemical oxygen demand (COD), total suspended solids (TSS), dissolved oxygen (DO), biochemical oxygen demand (BOD), and chemical oxygen demand (COD) were performed according to Winkler’s method (Strickland and Parsons 1972). Nutrients including chlorides (CL^−^), nitrite (NO_2_^−2^-N), nitrate (NO_3_^−1^-N), ammonium (NH_4_^+^-N), total nitrogen (TN), total phosphorus (TP), total dissolved solids (TDS), sulfides(S^−2^), and total hardness (TH) were measured in accordance with the standard procedures established by the American Public Health Association (APHA 2005).

### Microalgae cultivation

Two photoautotrophic media were used, Kühl medium and KC (Kessler and Czygan 1970), for microalgae cultivation (Table 1). The microalgal cultures were incubated at 25 ± 2 °C under a constant fluorescent light of 40 µmolm^−2^ s^−1^ and were constantly supplied with sterilized filtered air to prevent biomass from glomming at the flask’s bottom. The experiments were conducted in triplicate using 1000 mL Erlenmeyer flasks filled with 500 mL of culture medium and each inoculated with 10% of a vigorously growing microalgal culture.

**Table 1:** Composition of KC and Kühl media used for the microalgal cultivation

Chemical compounds	Concentration (mg /L)	
	KC	Kühl
KNO_3_	810	1011.1
NaCl	470	-
NaH_2_PO_4_.H_2_O	470	621
NaHPO_4_.2H_2_O	360	89
MgSO_4_.7H_2_O	250	246.5
(NH_4_)_6_Mo_7_O_24_.4H_2_O	0.2	0.0125
CaCl_2_.2H_2_O	15	14.7
ZnSO_4_.7H_2_O	0.2	0.287
H_3_BO_3_	0.5	0.061
MnCl_2_.4H_2_O	0.5	-
EDTA	8	-
Na_2_-EDTA	-	9.3
MnSO_4_.H_2_O	-	0.169
CuSO_4_.5H_2_O	-	0.0025
FeSO_4_.7H_2_O	6	6.95

### Cultivation of microalgae in wastewater mixed with synthetic medium

Areated wastewater (AWW) samples were collected and settled for 3 h at room temperature and the supernatant was used after sterilization in algal cultivation dilutions. The two isolated microalgae were grown in undiluted (100%) and diluted (25%, 50%, and 75%) AWW. Dilutions were made by adding the required amounts of AWW and KC medium. The experiments were conducted in triplicate in 2 L Erlenmeyer conta filled with 1 L of culture medium (each flask was stopped with cotton plugs and sterilized in autoclave at 121 ℃,1.5 bar for 20 min) after sterilization and cooling culture medium inoculated with 10% of exponentially growing microalgal culture. Each dilution was cultivated under the same cultivation condition of the “Microalgae cultivation” section. Microalgae grown in KC medium under the same growth conditions were sustained as the control.

### Studying the effect of different salt stress on algal performance

For improving the algal growth and lipid productivity, different NaCl concentrations ranging from 1.73 to 3.23 g/L were employed to modify the TDS levels from 2000 to 3500 mg/L. TDS levels of the modified medium were determined using a HACH TDS meter (model: sensION™ + EC71).

### Growth measurements

Algal growth was determined by optical density (OD) at 680 nm (Markle et al. 2000), at two days intervals using SHIMADZU UV-2401PC, Japan spectrophotometer. Dry weight (DW) was monitored by gravimetry. A linear correlation between OD680 and dry weight was determined for both algal strains:

1 $$\begin{array}{cc}OocystispusillaDW \left(\frac{g}{L}\right)= 0.258\left(OD680\right)+ 0.9623& {R}^{2} = 0.93\end{array}$$

2 $$\begin{array}{cc}ChlorococcusinfusionumDW \left(\frac{g}{L}\right)= 0.4756\left(OD680\right) + 0.3792& {R}^{2} = 0.99\end{array}$$

Biomass productivity was calculated according to Eq. (3)

3 $$\mathrm{Biomass\; productivity} (g DW/L /d)= ({DW}_{\mathrm{E}} -{DW}_{\mathrm{i}})/\Delta T$$

where, *DW*_E_ (mg/L) and *DWi* (mg/L) represent the algal dry weight at the initial and the end of exponential growth phase, respectively, while *ΔT* is the time difference.

### Estimation of lipid content for the isolated species

The lipid content was extracted by mixing a 100 mg of algal pellet with 20 mL chloroform: methanol (2:1, v/v) (Folch) and incubating overnight at 25 ± 2 °C in a shaking incubator. The mixture was subjected to centrifugation at 1500 × g for 10 min. Following centrifugation, the supernatant was carefully collected, and the remaining residue underwent two additional extraction cycles. The solvent was mixed with 2 mL of 1M NaCl. The lipid phase was collected and left to evaporate, and the total lipid content was estimated gravimetrically. Lipid productivity (LP) was calculated using Eq. (4):

4 $$\mathrm{Lipid\; productivity }(g DW/L /d) = ({LC}_{E} -{LC}_{i})/\Delta T$$

where *LC*_*E*_ and *LC*_*i*_ are the lipids content at the ending and initial of the exponential phase, respectively, and ΔT is the time difference.

### Fatty acid analysis

Next to lipid extraction, the transesterification method was carried out according to Radwan (1978) in which 50 mg of lipid was mixed with 5 mL of methanolic sulphuric acid mix (1:100 v/v sulphuric acid (98%) and methanol (100%) and 2 mL of benzene. The mix was heated in the water bath (WB) for 90 min at 90 ℃. Once cooled, the procedure involved adding 8 mL of distilled water and 5 mL of petroleum ether to the mixture, followed by thorough mixing. The ethereal oil layer containing fatty acid methyl esters (FAME) was separated and evaporated. This resulting material was utilized for gas–liquid chromatography. FAMEs were resuspended in 20 µL of acetonitrile and subjected to analysis using an HP-6890 gas–liquid chromatograph equipped with an HP-5 column (30m × 0.32 mm × 0.25 µm) and a flame ionization detector (FID). The temperature program for the chromatograph began at 140 °C for 3 min, followed by an increase at a rate of 1.5 °C per minute until it reached 187 °C. Subsequently, the temperature was raised at a rate of 5 °C per minute until it reached 220 °C, and this temperature was maintained for 5 min. The detector operated at 250 °C, and the injector temperature was set at 220 °C. The injection was performed in a splitless mode using one µL of FAMEs, and the carrier gas (nitrogen) flowed constantly at a rate of 1 mL/ min.

### The assessment of fuel properties for the generated algal fatty acid profiles

The assessment of fuel properties for the generated algal fatty acid profiles involved theoretical calculations of various biodiesel properties. These properties encompassed the average degree of unsaturation (ADU %), cetane number (CN), iodine value (IV, g I2.100/g oil), kinematic viscosity (υi, mm2/s), specific gravity (*ρ*), cloud point (CP, ℃), saponification value (SV, mg KOH/g), long chain saturation factor (LCSF, wt %), higher heating value (HHV), and cold filter plugging point (CFPP, °C). These calculations were performed using Eqs. (5) through (13), as described in studies by

5 $$\mathrm{ADU }= \Sigma N \times Mf$$

where *N* represents the number of carbon–carbon double bonds in each fatty acid, and Mf is the mass fraction of each fatty acid.

6 $$\upsilon i = -0.6313 ADU + 5.2065$$

7 $$\rho = 0.0055 ADU + 0.8726$$

8 $$CP = -3.356 ADU + 19.994$$

9 $$CN = -6.6684 ADU + 62.876$$

10 $$IV = 74.373 ADU + 12.710$$

11 $$LCSF = (0.1 \times C16:0) + (0.5 \times C18:0)$$

12 $$CFPP = (3.1417 \times LCSF) - 16.477$$

where C16:0, C18:0, C20:0, C22:0, and C24:0 is the percentage of the subsequent fatty acids.

13 $$Y = 117.9295 /X + 2.5905 (0<100)$$

where *X* represnts the content of linoleic and linolenic acids (wt %) (0 < *X* < 100), and *Y* is the oxidation stability per hour.

### Statistical analyses

The results are presented as the mean of three replicates, along with the standard deviation (SD) indicated as ± . To determine the significance of the data, a statistical analysis was conducted using a one-way analysis of variance (ANOVA) with a significance level set at *p* ≤ 0.05. The statistical analyses were performed using the SPSS software (IBM, version 22), and Duncan’s multiple range tests were applied for post hoc analysis. This rigorous statistical approach helps to assess and compare the data effectively.

## Results and discussion

### Water characteristics

The characteristics of the municipal wastewater used as a cultivation medium are detailed in Table 2. The wastewater had a moderate pH of 7.35 and a temperature of 26.54 °C. Dissolved oxygen was low at 1.05 ppm, while BOD and COD levels were elevated at 179.23 and 304.66 ppm, respectively. Turbidity was 9.67 NTU, with total dissolved solids (TDS) of 1500 ppm and total suspended solids (TSS) at 195 ppm. The wastewater was high in chlorides (1520.69 ppm) and alkalinity (600.72 ppm). Nutrient levels included sulfur compounds at 2.05 ppm, nitrite at 36.25 ppm, nitrate at 6.02 ppm, and phosphate at 47.02 ppm. This composition provided nutrients to support algal growth while requiring acclimation to the elevated organic matter. Municipal wastewater, also known as domestic wastewater, is the wastewater discharged from households, including bathrooms, laundry rooms, and kitchens. When compared to various other types of wastewater, municipal wastewater contains relatively lower content of nitrogen (ranging from 15 to 90 mg/L), phosphorus (ranging from 5 to 50 mg/L), and archetypally exhibits a reduced concentration of chemical oxygen demand (approximately 300 mg/L) (You et al. 2022). Due to these characteristics, municipal wastewater is often considered suitable for the cultivation of microalgae.

**Table 2:** Some wastewater parameters used in this study

Parameters	Value
Temperature (℃)	26.54 ± 0.22
pH	7.35 ± 0.18
DO (ppm)	1.05 ± 0.17
BOD (ppm)	179.23 ± 24.40
COD (ppm)	304.66 ± 23.71
Turbidity (NTU)	9.67 ± 0.15
TDS (ppm)	1500.02 ± 43.16
TSS (ppm)	195.33 ± 41.84
Chlorides (ppm)	1520.69 ± 65.21
Alkalinity (ppm)	600.72 ± 11.29
S^−2^ (ppm)	2.05 ± 0.17
NO_2_ (ppm)	36.25 ± 1.71
NO_3_ (ppm)	6.02 ± 0.22
SO_4_ (ppm)	0.31 ± 7.38
PO_4_ (ppm)	47.02 ± 3.27

### Microalgae identification

Our primary objective is to uncover and characterize novel indigenous microalgal strains that have naturally adapted to thrive in the specific wastewater conditions present in the local environment. By systematically screening microalgae from the target waste stream, we can pinpoint species that have undergone optimization processes specifically tailored to this unique ecological niche. Moreover, the use of native strains alleviates concerns related to the potential introduction of invasive foreign species into the ecosystem. In the course of this study, we successfully isolated and identified two native microalgae strains. These identifications were primarily based on morphological characteristics. The isolated microalgae have been identified as follows: *Oocystis pusilla* Hansgirg 1890: This species exhibits a colonial, oval form, with sizes ranging from 15 to 30 µm Fig. S1, A and *Chlorococcus infusionum* (Turpin) Kützing 1833: Characterized by its single-cell structure, this species takes on a spherical shape, typically measuring between 15 and 40 µm Fig. S1, B. Both of these species belong to the Chlorophyta phylum.

This strategic focus on native microalgae strains enhances our ability to harness the unique adaptability and resilience of these organisms, which have evolved to thrive in the specific wastewater conditions found within our local ecosystem.

### Determination of the best growing medium

The choice of an appropriate growth medium for superior biomass and lipid production is based on the algae’s growth needs, the impact of the medium’s components on the concluding product quality, and the cost of the medium’s components (Abomohra et al. 2016). KC and Kühl media were used for the cultivation of the isolated microalgae. KC medium showed better algal growth relative to Kühl medium. The KC medium is suitable for the growth of both algae as it contains low nitrate and NaH_2_PO_4_.H_2_O content and higher levels of Na_2_HPO_4_.2H_2_O, MgSO_4_.7H_2_O, and (NH_4_)_6_Mo_7_O_24_.4H_2_O as sources of phosphorus, magnesium, and molybdenum, respectively, compared to other growth media. However, the presence of NaCl in the KC medium can lead to alterations in water salinity, which, in turn, may have notable effects on the growth, metabolism, and photosynthesis of microalgae. The salt concentration in the culture medium is a critical factor to consider when studying microalgal physiology and biochemistry, as it can influence various cellular processes and ultimately impact research outcomes (Abomohra et al. 2020).

**Fig. 1:**
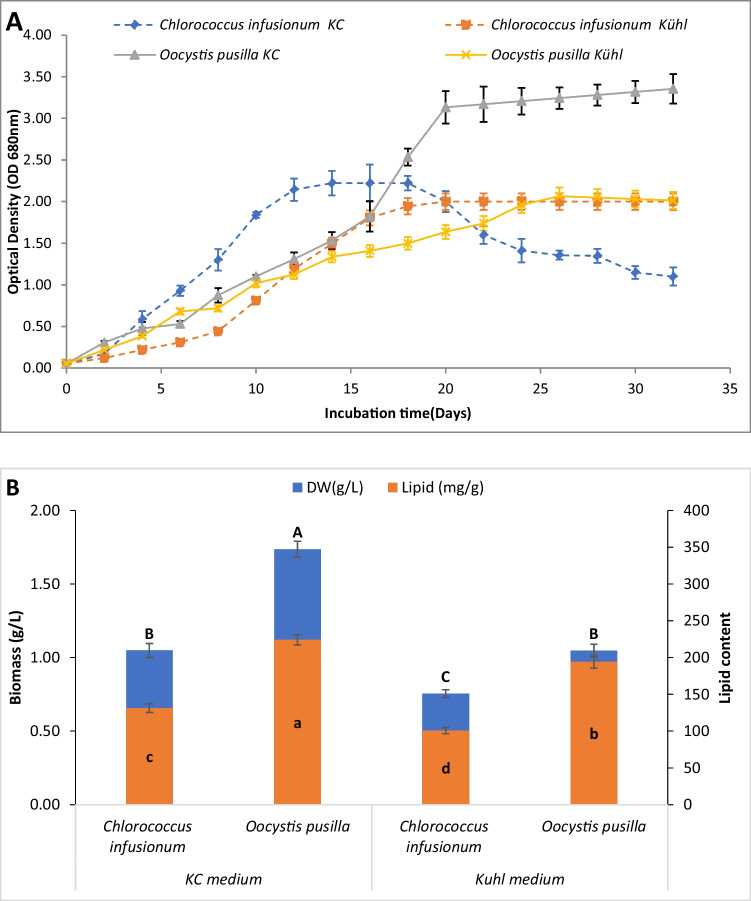
Growth curve (**A**) and biomass and lipid content (**B**) of *O. pusilla* and *C. infusionum* using KC and Kühl media. Different letters in each plotted series indicate significant differences at *p* < 0.05 using Duncan’s mean-separation test

On the other hand, *C. infusionum* showed low cell density compared to *O. pusilla* (Fig. 1A). The overall higher OD values achieved by *O. pusilla* compared to *C. infusionum* correlate with its higher biomass reported in Fig. 1B. The timing of the OD decrease varies between species and media types, likely reflecting differences in nutrient availability and uptake kinetics. KC medium supported a later peak and decline for *O. pusilla,* possibly due to more controlled nutrient release from the defined composition. The earlier peak and crash in KC cultures by *C. infusionum* suggests faster nutrient depletion in this complex medium, which may be due to the higher metabolism of *C. infusionum* rapidly exhausting nutrients in KC. It was observed that *O. pusilla* has a relatively long exponential phase compared to *C. infusionum*, extending to the 20th and 24th day in KC and Kühl media, respectively. This may be related to the nature and form of each species since colonial *O. pusilla* may exhibit different growth patterns compared to single-celled C. infusionum due to factors like cell–cell interactions, nutrient uptake, and light utilization. The shared sheath of *O. pusilla* may provide benefits like retaining nutrients and metabolites. The peak algal dry weight (1.73 g/L) was recorded in *O. pusilla* cultivated on KC medium on the 20th day, while only 1.047 g/L on Kühl medium on the 24th day in the stationary phase*.* The same trend was recorded in lipid content, where the most lipid content (224.17 mg/g) was demonstrated in *O. pusilla cultivated* on KC medium (Fig. 1B). Accordingly, KC medium was selected for the next experiment.

The growth phase of algal culture can affect the fatty acids profiles and lipid content. In some species, lipid content improves during the stationary growth phase, including *O. pusill*a and *C. infusionum*. The observed changes in microalgal physiology and lipid content in could be attributed to a shift in lipid metabolism. This shift may involve transitioning from synthesizing membrane lipids to storing neutral lipids. This metabolic change is likely prompted by the depletion of crucial nutrients, such as nitrogenous compounds required for protein synthesis and phosphate-containing compounds necessary for the formation of phospholipids, which are key structural components of cell membranes. As a result, the microalgae may prioritize the accumulation of neutral lipids, potentially for energy storage or other metabolic purposes, in response to the nutrient limitations induced by the salinity changes. (Xu et al. 2020; Chen and Wang 2021). Bigogno (2002) also found a similar increase in total fatty acid content and triacylglycerols in *Parietochloris incise* during the stationary phase, from 43% in the logarithmic phase to 77%.

### Biomass and lipid production in the wastewater mixture

**Fig. 2:**
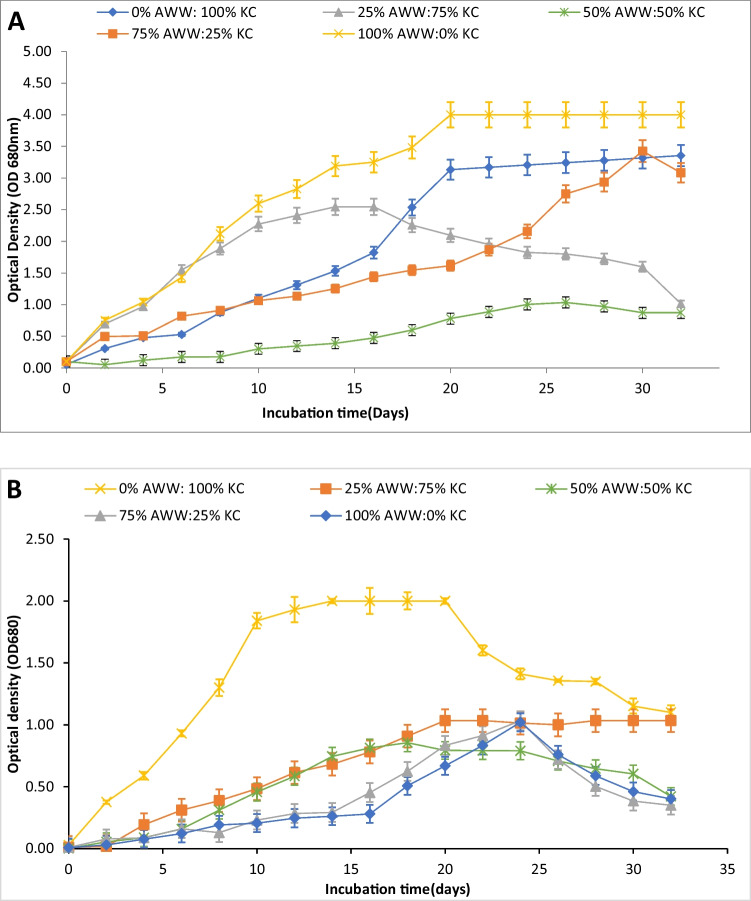
Growth curve of (**A**) *O. pusilla* and (**B**) *C. infusionum* at different ratios of aerated wastewater (AWW) to KC medium concentrations

Different concentrations (0, 25, 50, 75, and 100%) of KC medium were used mixed with AWW to cultivate both isolates. The tested microalgae showed different patterns regarding the ability to grow in AWW (Fig. 2). Results showed that *O. pusilla* was more adapted to grow in AWW than *C. infusionum.* In *O. pusilla,* 100% AWW (0%KC) led the maximum cell density reaching 4 OD (Fig. 2A) with a dry weight of 1.96 g/L at the 20th day of cultivation (Table 3), while *C. infusionum was* seriously affected by wastewater, and the highest cell density of 2 was recorded in 100% KC (Fig. 2B) with 1.05 g /L and decreased by increasing AWW concentration (Table 3). On the other hand, growing *O. pusilla* on 0% AWW (100% KC control) represents in the second order, where the highest OD and biomass were 3.35 and 1.73 g/L. When the KC medium was diluted with AWW, *O. pusilla*’s growth decreased, showing moderate growth in a medium composed of 25% AWW and 75% KC medium. This condition led to moderate biomass concentrations over 15 days of cultivation, followed by a decline in density after 15 days (Fig. 2A). The KC medium likely provided essential nutrients and optimized conditions that initially supported *O. pusilla*’s growth. However, the diluted KC medium reduced growth slightly at 25% AWW compared to 100% KC medium.

**Table 3:** Biomass and lipid content of *O. pusilla* and *C. infusionum* at different KC medium concentrations

*Treatment*	*DW (g/L)*		*Lipids (mg/g dw)*	
	*O. pusilla*	*C. infusionum*	*O. pusilla*	*C. infusionum*
*0% KC*	1.96 ± 0.01^a^	0.78 ± 0.02^c^	303.42 ± 16.39^a^	95.78 ± 0.01^c^
*25% KC*	1.62 ± 1.37^b^	0.67 ± 0.05^c^	262.83 ± 42.60^b^	91.46 ± 0.02^c^
*50% KC*	1.06 ± 0.02^d^	0.56 ± 0.02^d^	220.50 ± 65.57^c^	112.04 ± 0.02^b^
*75% KC*	1.44 ± 0.01^c^	0.87 ± 0.01^b^	211.67 ± 7.64^d^	86.77 ± 0.01^d^
*100% KC*	1.73 ± 0.01^ab^	1.05 ± 0.04^a^	224.17 ± 13.92^c^	131.32 ± 0.03^a^

Different superscript letters of the same strain in the column indicate significant differences at *p* < 0.05 according to Duncan’s mean-separation test

The growth of *O. pusilla* showed a decreasing trend initially as the wastewater concentration increased from 25 to 50%, but then increased with further increases in wastewater concentration up to 100% (Fig. 2A). The initial decline in growth in the 50% wastewater experiment compared to 25% wastewater might be due to the low optimum nutrient levels and the different components of wastewater versus the nutrient level at the 25% experiment. However, in experiments with 75% and 100% wastewater, *O. pusilla* likely preferred the wastewater conditions of low nitrate and phosphate content compared to the KC medium, allowing it to thrive and achieve higher growth. The increasing growth with higher wastewater concentrations in individual experiments indicates *O. pusilla’s* ability to utilize low mixed nutrient loads such as nitrite, nitrate, and phosphate, a useful trait for cultivation using wastewater as a growth medium.

A similar tendency was detected in terms of lipid content, with the highest biomass also showing the highest lipid content. The most significant lipid accumulation of 303.42 mg/g dw was observed in *O. pusilla* cultivated in 100% AWW, while *C. infusionum* showed a maximum lipid accumulation of 224.17 mg/g dw in 100% KC medium (Table 3). *O. pusilla* demonstrated a remarkable adaptability to highly concentrated municipal wastewater.

Several investigations have demonstrated that microalgae have the capacity to effectively utilize phosphorus, nitrogen, and organic substances from different wastewater sources. This process not only helps in the purification of wastewater but also leads to the generation of biomass. These wastewater sources encompass municipal, agro-industrial, industrial, and livestock wastewater (Srinuanpan et al. 2020; Srimongkol et al. 2022). For example, *Tribonema minus* has been found to thrive in simulated butyric acid wastewater and its biomass and lipid productivity accomplished the superior of 223.80 and 108.16 mg/L/day, respectively (Wang et al. 2023a, b), while halophilic oleaginous microalgae *Nannochloropsis oculata* and *Tetraselmis chuii* have demonstrated survival in industrial wastewater and the highest biomass and lipid productivity were documented using a ratio of 75% industrial WW:25% F/2 medium (Essa et al. 2018). Additionally, *Chlorella pyrenoidosa* has been shown to survive in piggery wastewater (Wang et al. 2012). A study by Shu et al. compared the growth and lipid content of three microalgae species (*Chlorella zofingiensis, Chlorella* sp., and *Scenedesmus* sp) in BG11 medium and raw dairy wastewater. The study found that all three microalgae species grew faster and produced more lipids in dairy wastewater than in synthetic medium (Shu et al. 2018). Another study compared the growth and lipid yield of three green microalgae, *Scenedesmus obliquus Chlamydomonas reinhardtii*, and *Monoraphidium braunii,* cultivated in sewage water and synthetic medium. It was observed that the growth rates of both *S. obliquus* and *M. braunii* were substantially reduced when cultivated in wastewater compared to the control group cultivated in synthetic medium. In contrast, *C. reinhardtii* displayed the most significant growth rate when grown in sewage wastewater (El-Sheekh et al. 2023).

Conversely, the lipid contents and productivity of *C. reinhardtii, M. braunii,* and *S. obliquus* were enhanced when cultivated in sewage wastewater as opposed to synthetic medium (El-Sheekh et al. 2023). This indicates that different microalgae species may have varying responses to wastewater conditions. As demonstrated in this study, *O. pusilla* appears to excel in wastewater cultivation compared to *C. infusionum*. This variability in behavior between these two microalgae species can be attributed to their distinctive abilities, not only to survive and flourish in wastewater but also to effectively harness its nutrients. These differences in adaptation and nutrient utilization strategies make certain microalgae, like *O. pusilla*, well-suited for wastewater-based cultivation, presenting valuable opportunities for applications in biofuel production.

The lipid productivities achieved by *O. pusilla* and *C. infusionum* are comparable to those reported for other high-oil-producing microalgal species grown on wastewater. A recent study isolating oleaginous *Oocystis* sp. and *Chlorococcum* sp. from dairy farm wastewater found *Oocystis* sp. had superior lipid content (24.70% dw) compared to *Chlorococcum* sp (18.68% dw) (Sun et al. 2018). The elevated lipid content observed in *O. pusilla* cultivated in 100% AWW (secondary treated municipal wastewater) may be attributed to variations in biotic stress responses among different algal species. Additionally, it could result from a notable deficiency in essential elements, particularly nitrogen, within the secondary treated municipal wastewater. These conditions might have triggered a metabolic response in *O. pusilla* that prioritizes lipid accumulation, possibly as an adaptive strategy to cope with nutrient limitations and environmental stressors present in the wastewater. This nitrogen scarcity led to an increased accumulation of lipids (Rodríguez-Palacio et al. 2022). These findings are consistent with prior research by Elshobary et al. (2019) and El Shafay et al. (2021), who demonstrated that green microalgae (*Micractinium reisseri*) and blue-green algae (*Anabaena variabilis* and *Nostoc muscorum*) tend to amass superior lipid content when subjected to nitrogen-low conditions.

However, *C. infusionum* cannot survive in such highly polluted conditions in 100% AWW. This result could be explained by *C. infusionum* having different nutrient requirements or metabolic pathways compared to *O. pusilla*, allowing *O. pusilla* to achieve better biomass and lipid yields. The nutrient content, salt, or other constituent concentrations in the 100% wastewater may have exceeded the tolerance levels of *C. infusionum*, inhibiting its growth. *O. pusilla* appears more tolerant of harsh wastewater conditions. The difference in behavior of both microalgae is attributed to the ability of each species to survive and grow in wastewater as well as utilize its nutrients efficiently (Sforza et al. 2020). As a result, *O. pusilla* cultivated in 100% AWW was used for the remainder of the study.

### Effect of salt stress on the growth and lipid content of *O. pusilla*

**Fig. 3:**
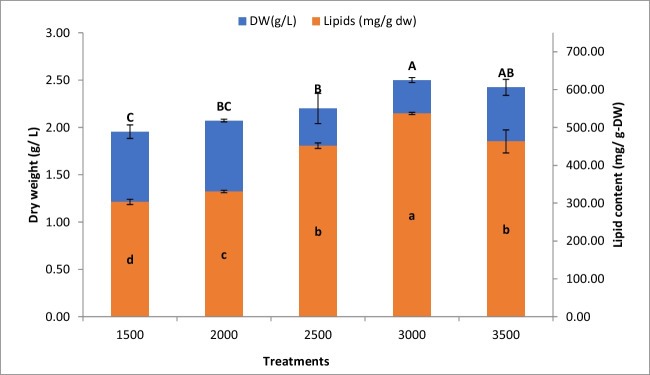
Biomass and lipid content of *O. pusilla* cultivated on 100% AWW supplemented with different TDS (ppm). Different letters in each plotted series indicate significant differences at *p* < 0.05 using Duncan’s mean-separation test

Four concentrations of NaCl were used to study their impact on the growth performance and lipid production of *O. pusilla.* Figure 3 shows that NaCl at a TDS concentration of 3000 ppm showed the maximum biomass and lipid content of 2.5 g/L and 536.88 g/L, with an improvement of 27.55% and 76.94% compared to the control (1500 ppm). Salinity stress has yielded lipid storage in microalgae (Venkata Mohan and Devi 2014; El-Sheekh et al. 2019). When exposed to salinity stress, microalgae undergo various physiological changes to adapt to their new environment. One of these changes is an improvement in lipid accumulation. This is because lipids play an essential role in cellular membrane composition and function, and high salt concentrations can disrupt the integrity of the membrane. As a result, the microalgae increase their lipid production to repair and protect their cellular membranes. When biomass and lipid productivities were studied, yields showed higher values in the salinity condition of 3000 ppm (113.33 and 52 mg/L/d, respectively) (*p* < 0.01) because of higher growth and higher lipid content (when compared to other TDS concentrations) (Table 4). Assuming high biomass and lipid productivities are desired for the utilized biofuel production, TDS in the upper level at 3000 ppm of high growth of *O. pusilla* would maximize lipid production. The hypersaline levels at 3000 ppm of *O. pusilla* grown on 100% AWW could likely enhance *O. pusilla’s* fatty acid profile and transesterification to biodiesel.

**Table 4:** Biomass and lipid productivities of *O. pusilla* grown on 100% AWW at different concentrations of TDS (ppm) compared to 100% KC

TDS CONC. (PPM)	BIOMASS PRODUCTIVITY (MG /L /D)	LIPID PRODUCTIVITY (MG /L /D)
KC	88.43 ± 1.24^d^	20.32 ± 0.22^c^
1500	93.61 ± 3.34^c^	23.68 ± 0.62^c^
2000	98.49 ± 2.59^c^	18.76 ± 0.40^d^
2500	103.89 ± 1.33^b^	43.63 ± 0.99^b^
3000	113.33 ± 2.10^a^	52.62 ± 0.97^a^
3500	111.74 ± 1.74^a^	47.70 ± 0.80^b^

Different superscript letters of the same strain in the column indicate significant differences at *p* < 0.05 according to Duncan’s mean-separation test

### Fatty acid profile *O. pusilla*

The fatty acid content analysis for *O. pusilla* cultivated in 100% AWW with a total dissolved solids (TDS) of 3000 ppm, where the highest biomass and lipid productivity were observed, revealed some interesting findings. In Table 5, it is shown that the saturated fatty acid (SFA) content was notably high, at 41.76%, followed by monounsaturated fatty acids (MUFA) and polyunsaturated fatty acids (PUFA).

Among the detected fatty acids with chain lengths ranging from C12 to C18, C16:0 stood out as the dominant saturated fatty acid, constituting 30.38% of the total fatty acids. On the other hand, C18:1 was the predominant fatty acid in the monounsaturated category, while C18:2 was the dominant one in the polyunsaturated category (Table 5).

**Table 5:** Fatty acid content and fatty acid methyl esters (FAMEs) proportions of *O. pusilla* grown on 100% AWW supplemented with different TDS (ppm)

*Fatty acids*	*Type*	*Content (%)*
*Dodecanoic acid*	C12:0	2.25
*Methyl tetradecanoate*	C14:0	2.46
*Methyl palmitate*	C16:0	30.38
*Methyl stearate*	C18:0	6.67
*9-Hexadecenoic acid*	C16:1	6.57
*9-Octadecenoic acid*	C18:1	16.78
*9,12-Octdecadienoic acid*	C18:2	8.63
*9,12,15-Octadecatrienoic acid*	C18:3	4.84
*SFAs*		41.76
*MUFAs*		23.35
*PUFAs*		13.47

Interestingly, *O. pusilla* primarily produced fatty acids with chain lengths of C16 and C18, accounting for a significant portion, specifically 73.87%, of the total fatty acid methyl esters (FAME). Furthermore, the algae displayed low levels of unsaturation in their fatty acids, and no polyunsaturated fatty acids with four or more double bonds were detected. This fatty acid composition and structure are considered favorable for biodiesel production, indicating the potential suitability of *O. pusilla* as a feedstock for biodiesel production due to its high content of saturated and monounsaturated fatty acids.

The findings from the current study align with those of Matos et al. (2015) and Abomohra et al. (2020). Their research explored the impact of desalination concentrate on the fatty acid profile of *N. gaditana* and *Dunaliella salina* KSA-HS022, respectively. They observed a similar trend, where an increase in the concentration of desalination concentrate led to an increase in saturated fatty acids (SFAs) at the expense of polyunsaturated fatty acids (PUFAs). This consistency in results across different studies underscores the significance of environmental factors, such as salinity or nutrient availability, in influencing the fatty acid composition of microalgae.

### Biodiesel quality

This study calculated and compared the essential qualitative characteristics of biodiesel produced from each treatment to the standards outlined in the American (ASTM D6751) and European (EN 14214) international guidelines, as illustrated in Table 6. The results indicate that the kinematic viscosity (υi), cetane number (CN), iodine value (IV), cloud point (CP), specific gravity, and higher heating value (HHV) values met the criteria set by both ASTM D6751 and EN14214 standards.

**Table 6:** The estimated properties of biodiesel derived from *Oocystis pusilla* cultivated on 100% AWW at TDS 3000 ppm in comparison with the international standards

Biodiesel parameter	Units	*O. pusilla*	ASTMD6751-02	EN14214
Average degree unsaturation (ADU)	−	0.55	−	−
Kinematic viscosity (υi)	Mm/s	4.86	1.9–6.0	3.5–5.0
Cetane number (CN)	−	59.2	≥ 47	≥ 51
Iodine value (IV)	g I_2_100/g Oil	53.71	NA	120 ≥
Cloud point (CP),	°C	12.63	−	> 4
Specific gravity (ρ)	g/m^3^	0.88	0.878	0.86–0.90
Higher heating value (HHV)	MJ/kg	39.5	NA	−
Long chain saturation factor (LCSF)	−	6.37	−	−
Cold filter plugging point (CFPP)	°C	3.55	NA	≤ 5 ≥ –20
Oxidation stability (Y)	h	11	−	≥ 6
C18:3	wt%	0.05	−	≤ 12

Furthermore, the average degree of unsaturation (ADU) of *O*. *pusilla* FAMEs was determined to be 0.55, which indicates a relatively low level of unsaturation. It is well-established that a low degree of unsaturation is a crucial factor in determining the quality of biodiesel. This ADU value is consistent with various ADU values observed in biodiesel derived from different microalgae sources, suggesting that *O. pusilla*-derived biodiesel aligns with the established standards and quality characteristics of biodiesel in this context. (Ashour et al. 2019; Elshobary et al. 2019, 2022; El Shafay et al. 2021) or macroalgae (Elshobary et al. 2020; Osman et al. 2020). The CN measures the ignition efficiency of biodiesel, increasing as the saturation level of fatty acids increases (Karpagam et al. 2015). Saturated long-chain esters such as methyl palmitate C_17_H_34_O_2_ and methyl stearate C_19_H_38_O_2_ have high cetane numbers (Knothe 2016). *O. pusilla* obtained biodiesel rich in these two fatty acids methyl esters 37.05%. Following international standards such as ASTM D6751-02, which recommends a minimum cetane number (CN) of 47, and EN14214, which suggests a CN of at least 51, it was found that the biodiesel produced by O. pusilla exhibited a favorable CN value of 59.2. This value ensures that the engine can initiate combustion quickly and quietly, indicating efficient ignition performance and minimal emissions of nitrogen oxides (NOx) (Ashokkumar et al. 2015; Sharma et al. 2016). It is important to note that the lowest IV value indicates the highest saturation level of methyl esters and offers high stability against oxidation (Srinuanpan et al. 2018), in contrast to the CN value. Our results were steady with different biodiesels produced from diverse green microalgae (Wang et al. 2013, 2023b; Song et al. 2013; Elshobary et al. 2019). Biodiesel generally has a higher cloud point compared to petroleum diesel fuel. In the current study, the cloud point (CP) of *O. pusilla* was 12.63 °C, which enhances its suitability for use in low-temperature conditions. Similarly, the recommended biodiesel strain, *Tribonema minus*, also showed 12.50 °C cloud point values when cultivated on synthetic butyric acid wastewater (Wang et al. 2023b). The higher heating value (HHV) refers to the quantity of heat generated by completely burning a specific amount of fuel. According to previous studies, the HHV values obtained in the current study were deemed satisfactory (Zhang et al. 2018; Elshobary et al. 2022) that is higher than that recorded in *Oocystis submarina* ( 29.47 and 32.98 MJ kg^−1^) under 100% and 50% of F/2 synthetic medium, respectively (Hawrot-Paw et al. 2021). This variation could be due to differences in the fatty acid composition of both species and the growth conditions, as algae have the ability to alter their fatty acid profile as well as morpho-anatomical and biochemical characterization in facing environmental stress (Barakat et al. 2021; Senousy et al. 2023; Ismail et al. 2023). Another parameter calculated was LCSF, which determines the fuel’s cold flow properties and is usually detected by the cold filter plugging point (CFPP). The information in Table 5 clearly shows *O. pusilla*’s long chain saturation factor (LCSF) value, which was determined to be 6.37. Additionally, the calculated cold filter plugging point (CFPP) value was found to be 3.55 °C. It is worth noting that CFPP values can vary depending on the level of unsaturation in the methyl ester. As per the findings of Ramos et al., lower CFPP values are preferred, and this study recorded a CFPP value of 3.55 °C, which is in line with the desirable characteristic of having a lower CFPP for biodiesel (Ramos et al. 2009) and falls within the EN14214 range (≤ 5 ≥ –20 °C). The oxidation stability of the biodiesel produced from *O. pusilla*, which indicates the formation of sediments, resins, and acids in the fuel, was found to be 11 h. This result meets the European standard (EN14214).

Based on these findings, it can be concluded that the cultivation of *O. pusilla* in the Hurghada aerated wastewater plant resulted in biodiesel that complies with both American standards (ASTM D6751-02) and European standards (EN 14214) in all key properties. This indicates the potential suitability of *O. pusilla*-derived biodiesel for use in accordance with these international standards.

### Implementation feasibility assessment

While this study does not provide an extensive feasibility analysis demonstrating the practical applications, it is possible to derive a reasonable assessment of the potential benefits of utilizing wastewater effluent as a growth medium for *O. pusilla* by incorporating data from this study. The results were specifically utilized to illustrate the impact of wastewater utilization on the cost of microalgal biomass production. However, it should be noted that these findings may not be directly applicable or reliable for future projects.

Davis et al. (2016) reported a fully feasible assessment of a 5000-acre commercial microalgal biomass production project. The total land area was about 7600 acres accounting for required facilities. The annual production capacity was 38 tons/acre/y with a cultivation productivity of 25 g/m^2^/d. Davis et al. (2016) concluded the production cost was $121.3 per tonne/hectare for 10-acre pond designs and recommended recycling nutrients to minimize the greenhouse gas footprint. In this context, water, ammonia, and nutrients could be replaced with wastewater as a growth medium for *O. pusilla*.

Based on the biomass production result of 2.5 g/L in 100% wastewater, the wastewater could substitute for conventional growth media, making the total production cost comparable despite little increased pretreatment costs. Based on observed biomass productivities, the projected annual biomass production could reach 25–30 tonnes/hectare utilizing *O. pusilla* grown on local municipal wastewater. With a lipid content of 536.8 mg/g DW at 3000 TDS, the projected annual oil yield is estimated at 13.4–16.1 tonnes/hectare. With estimated cultivation cost of $121.3 per tonne/hectare and extraction cost of $500–1000 per tonne, the production cost is projected to be around $0.25–0.5 per kg of oil. At current biodiesel prices of $1.5–3 per kg, this suggests strong potential profitability of the proposed system. Scaled up to a 100-hectare facility, total annual oil production could reach 1340–1610 tonnes, demonstrating potential scalability.

This simple assessment shows the potential for wastewater utilization, as it often exceeds surrounding industry and civilization regions. Excess wastewater spreading causes negative environmental impacts through nutrient leaching, eutrophication, and GHG emissions. Therefore, microalgae cultivation enables nutrient recycling and recovery from wastewater in a cost-effective manner.

## Conclusion

Cultivating microalgae in wastewater represents a sustainable and eco-friendly approach for biodiesel production while mitigating wastewater disposal’s environmental consequences. *O. pusilla* emerges as an auspicious choice for biodiesel production using wastewater as a growth medium, mainly due to its impressive biomass productivity and lipid yield. Moreover, elevating the total dissolved solids (TDS) level in the culture medium can substantially increase lipid yield. The fatty acid composition of the cultivated microalgae aligns with the stipulated values in American and European biodiesel standards, underscoring the feasibility of this method as a cost-effective and environmentally responsible means of generating biofuels. Our findings help fill knowledge gaps regarding growth kinetics, nutrient requirements, and salt tolerance for these specific strains. By elucidating the differences between these two species, our study contributes to the ongoing characterization of microalgae biodiversity for biofuel production. More research is needed to optimize the cultivation conditions and lipid extraction methods to improve the economic feasibility of this approach.

### Supplementary Information

Below is the link to the electronic supplementary material.

Supplementary file1 (DOCX 817 KB)

## Data Availability

All data generated or analyzed during this study are included in this published article.
