# Supplementary material for: Unlocking the potential of microalgae cultivated on wastewater combined with salinity stress to improve biodiesel production
Source: Environ Sci Pollut Res Int. 2023 Oct 21;30(53):114610–24. doi: 10.1007/s11356-023-30370-6 (PMC10663198; doi:10.1007/s11356-023-30370-6)
Supplement: Supplementary file 1 — Supplementary file1 (DOCX 817 KB) [file 11356_2023_30370_MOESM1_ESM.docx]

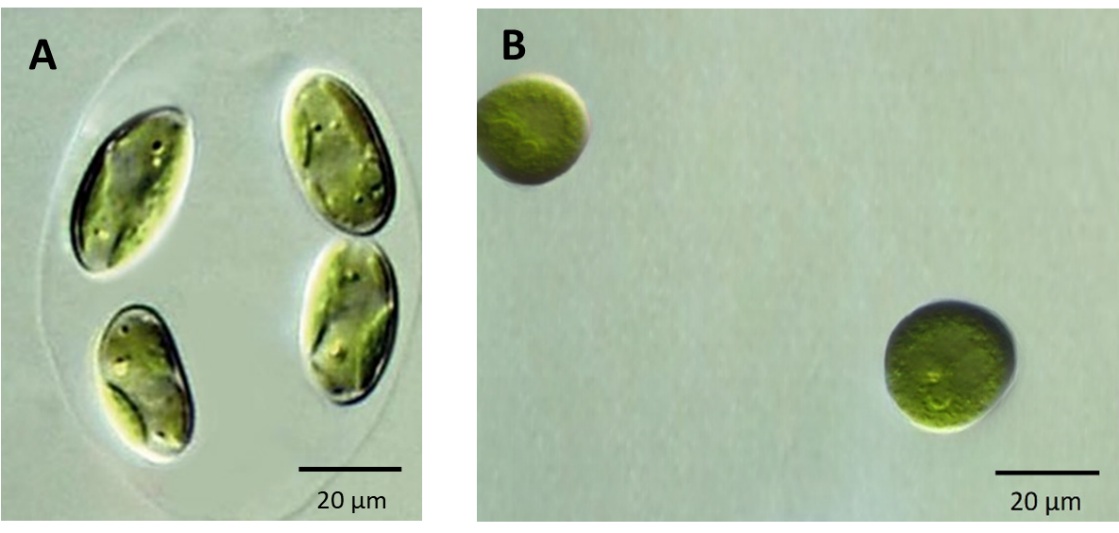


Fig. S1. Light microscopic images of cell morphology of the isolated alga *O. pusilla* (A). *C. infusionum* (B) (culture after 15 days), Scale bars: 20 μm.
